# Endoplasmic reticulum stress in the salivary glands of patients with primary and associated Sjögren’s disease, and non-Sjögren’s sicca syndrome: a comparative analysis and the influence of chloroquine

**DOI:** 10.1186/s42358-024-00430-7

**Published:** 2025-01-08

**Authors:** Graziela Vieira Cavalcanti, Fabiola Reis de Oliveira, Rafael Ferraz Bannitz, Natalia Aparecida de Paula, Ana Carolina Fragoso Motta, Eduardo Melani Rocha, John Chiorini, Hilton Marcos Alves Ricz, Denny Marcos Garcia, Maria Cristina Foss-Freitas, Luiz Carlos Conti de Freitas

**Affiliations:** 1 Department of Ophthalmology, Otolaryngology, Head and Neck Surgery, Ribeirão Preto Medical School, University of São Paulo, São Paulo, Brazil; 2 Department of Internal Medicine, Ribeirão Preto Medical School, University of São Paulo, São Paulo, Brazil; 3 Department of Stomatology, Public Health and Forensic Dentistry, Ribeirão Preto School of Dentistry, University of São Paulo, São Paulo, Brazil; 4 Adeno-Associated Virus Biology Section, National Institute of Dental and Craniofacial Research, National Institutes of Health, Bethesda, MD USA; 5 Division of Metabolism, Endocrinology, and Diabetes, Department of Internal Medicine, and Caswell Diabetes Institute, University of Michigan, Ann Arbor, MI USA; 6 Ophthalmology, Otolaryngology, Head and Neck Surgery Department, Ribeirão Preto Medical School, University of São Paulo, Av. Bandeirantes 3900, Monte Alegre, Ribeirão Preto, SP 14.040-900 Brazil

**Keywords:** Sjögren syndrome, Gene expression, Cross-sectional studies, Endoplasmic reticulum stress, Unfolded protein response

## Abstract

**Background:**

Endoplasmic reticulum stress (ERS) and the unfolded protein response (UPR) are adaptive mechanisms for conditions of high protein demand, marked by an accumulation of misfolded proteins in the endoplasmic reticulum (ER). Rheumatic autoimmune diseases (RAD) are known to be associated with chronic inflammation and an ERS state. However, the activation of UPR signaling pathways is not completely understood in Sjögren’s disease (SD). This study evaluated the expression of ERS-related genes in glandular tissue of patients with primary SD (pSD), associated SD (aSD) with other autoimmune diseases, and non-Sjögren sicca syndrome (NSS).

**Methods:**

In a cross-sectional study, minor salivary gland biopsies were obtained from 44 patients with suspected SD and 13 healthy controls (HC). Patients were classified as pSD, aSD, or NSS based on clinical, serological, and histological assessment. Histopathological analysis and mRNA expression analysis of genes associated with ERS and UPR (PERK, XBP1, ATF-6, ATF-4, CANX, CALR, CHOP, and BIP) were performed on the samples. Differences between groups (pSD, aSD, NSS, and HC) were assessed. The influence of chloroquine (CQ) on the ER was also investigated.

**Results:**

Twenty-eight SD patients showed increased expression of PERK (*p* = 0.0117) and XBP1 (*p* = 0.0346), and reduced expression of ATF-6 (*p* = 0.0003) and CHOP (*p* = 0.0003), compared to the HC group. Increased expression of BIP (*p* < 0.0001), PERK (*p* = 0.0003), CALR (*p* < 0.0001), and CANX (*p* = 0.0111) was also observed in the SD group compared to the NSS group (*n* = 16). Patients receiving CQ (*n* = 16) showed a significant increase in ATF-6 (*p* = 0.0317) compared to patients not taking the medication (*n* = 29).

**Conclusions:**

Altogether, the results suggest a greater activation of the ERS and UPR genes in patients with SD, especially in the pSD group. Antimalarial drugs, like CQ, used to treat RAD, may affect the ER function in exocrine glands.

**Supplementary Information:**

The online version contains supplementary material available at 10.1186/s42358-024-00430-7.

## Introduction

Sjögren disease (SD) is a rheumatic autoimmune disease (RAD) that affects exocrine glands, particularly salivary and lacrimal glands, and is characterized by lymphocytic tissue infiltration resulting in dry eye and dry mouth symptoms. The condition is classified either as a primary disease (pSD) or as an associated disease (aSD), i.e., when it overlaps other systemic RADs [1]. Patients with non-Sjögren *sicca* syndrome (NSS), although presenting dryness symptoms, do not have sufficient evidence to confirm the diagnosis of SD [2].

Autoimmune diseases (AID) have been associated with disruption of endoplasmic reticulum (ER) homeostasis and accumulation of unfolded proteins, a condition known as endoplasmic reticulum stress (ERS) [3]. Under regular circumstances, a group of molecular chaperones and folding enzymes act on the assembly of new proteins. Due to the overload induced by the proinflammatory state in AID, the unfolded protein response (UPR) is activated, transiently mitigating protein biosynthesis and increasing the ER protein folding capacity. Another group of chaperones, the ER-associated degradation (ERAD) pathway, contributes to the clearance of misfolded proteins by the cytosolic 26 S proteasome, together with the autophagy-lysosome pathway. This phenomenon activates transcription factors and the expression of chaperones and folding enzymes, promoting cell adaptation and restoring homeostasis. One of the first chaperones to be activated in the UPR is binding protein (BIP), which signals three other major transmembrane proteins – activating transcription factor 6 (ATF-6), protein endoplasmic reticulum kinase (PERK), and inositol-requiring enzyme 1α (IRE1α) – involved in transcriptional and translational regulation of genes [4].

**Fig. 1:**
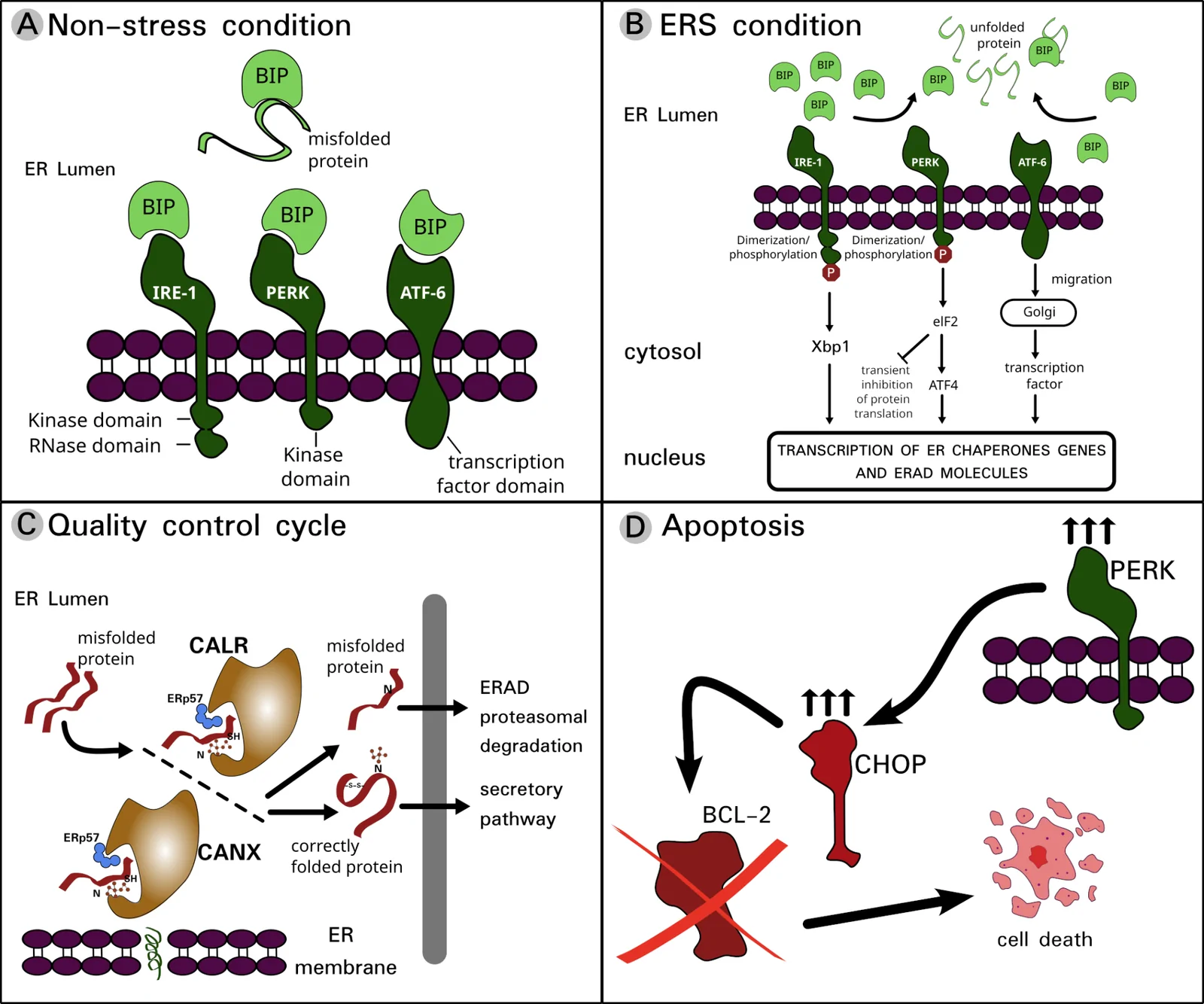
The endoplasmic reticulum stress (ERS) and the unfolded protein response (UPR) mechanisms in the cell. *Legend 1* Salivary glands have a high protein synthesis which increases unfolded protein levels in the endoplasmic reticulum (ER) lumen, which triggers ERS and UPR, and ultimately leads to reduction of secretion protein and dryness. The following pictures schematically represent these mechanisms. **A** In a non-stress condition, there are few or no misfolded proteins in the ER lumen, so BIP remains bound to the three principal ER membrane UPR sensors: IRE1α, ATF-6, and PERK. **B** Under ERS conditions like accumulation of misfolded proteins, oxidative stress, Ca^2+^ depletion, inflammatory cytokines, metabolic starvation, aging, heat, and pH variation, BIP dissociates from the UPR sensors. BIP dissociation from IRE1α or PERK causes its oligomerization and autophosphorylation. IRE1α catalyzes the splicing of XBP1. Activated PERK phosphorylates eIF2a that transiently attenuates protein translation and acts on ATF-4 to increase transcription of genes promoting cell survival under stress conditions. BIP dissociation from ATF-6 causes translocation of ATF-6 to the Golgi, where a transcription factor is released. These responses lead to activation of ERS signaling pathways increasing expression of ER chaperones and molecules associated with endoplasmic reticulum-associated protein degradation (ERAD). **C** Misfolded proteins connect to the CANX-CALR quality control cycle along with ERp57. When the substrate achieves its final conformation, the correctly folded protein leaves the ER lumen. Some proteins require multiple rounds of association with the chaperones, re-entering the CANX-CALR cycle. Terminally misfolded proteins are targeted for ERAD. **D** Activated by PERK, ATF-4 upregulates CHOP, which tips the ER toward homeostasis through the induction of several corrective genes, including XBP1 and chaperones. However, sustained PERK signaling is incompatible with survival. High levels of CHOP can inhibit the expression of antiapoptotic BCL-2 and induce apoptosis

Under ERS, ATF-6 promotes the expression of genes involved in protein folding and activates the ERAD pathway for protein degradation in the cytoplasm. Phosphorylated PERK, as a sensor of ERS, inhibits eIF2α activity and protein synthesis, thereby preventing further influx of polypeptides into the ER, and giving the cell extra time to fold the proteins already present in the ER lumen. In contrast, the translation of activating transcription factor 4 (ATF-4) is increased when the amount of active eIF2α is limited. The expression of ATF-4 increases C/EBP- homologous protein (CHOP), which tips the ER toward homeostasis through the induction of several corrective genes, including x-box protein 1 (XBP1), and drives the cell into a pro-apoptotic state. IRE1α also splices the mRNA of the transcription factor XBP1, which contributes to changes in the transcriptome [5, 6]. Although a temporary pause in protein translation due to eIF2α phosphorylation can be beneficial for cells under ERS, a prolonged block in translation from sustained PERK signaling leads to high levels of CHOP, which can inhibit the expression of antiapoptotic B-cell lymphoma 2 protein (BCL-2), causing cellular apoptosis [5, 7]. Calreticulin (CALR) and calnexin (CANX) support signaling pathways responding to stress [4]. Together, these components help homeostasis within the ER. The mechanisms of ERS and UPR are illustrated in Fig. 1.

In general, exocrine glands require high protein production, a process that may lead to ERS activation and apoptosis. In SD, it is already known that a hyperosmolar environment in dry eyes and the presence of interferon (IFN)-γ trigger ERS on the ocular surface, causing epitheliopathy [8]. Moreover, autophagy and ERS-induced apoptosis in human salivary gland cells relocate Ro/SSA and La/SSB autoantigens, rendering them immunogenic and initiating an adaptive immune response that involves T cell-mediated B cell production of autoantibodies and lymphocytic tissue infiltration [9]. In vitro and animal model studies have tested some small molecules and chemical chaperones (e.g. tauroursodeoxycholic acid) to reverse the process [10–13].

Chloroquine (CQ) is widely used for the treatment of patients with systemic lupus erythematosus (SLE) and SD [14, 15]. The drug inhibits lysosomal activity and autophagy, prevents MHC class II-mediated autoantigen presentation, and alters transcriptional activity that decreases cytokine production (as interleukin-6, TNF, and IFN-γ), toll-like receptors signaling and modulation of co-stimulatory molecules [16–18]. Novel insights into the mechanism underlying CQ effects indicate that the upregulation of CHOP may mediate CQ-induced extrinsic pathways and autophagy-dependent apoptosis [19]. Therefore, it is reasonable to consider that the effects of CQ on protein synthesis and ERS may contribute to clinical benefits in SD.

Although some studies have reported the association between ERS and SD [3, 20, 21], none have assessed whether there are differences between pSD, aSD, and NSS individuals. Additionally, neither the simultaneous performance of the three main UPR pathways nor the effect of CQ treatment on ERS has been evaluated to date. Thus, the present study aimed to examine the behavior of ERS in patients with pSD, aSD, SD (pSD + aSD), NSS, and healthy control (HC) groups, evaluating the expression of the genes BIP, PERK, XBP1, ATF-6, ATF-4, calreticulin (CALR), calnexin (CANX), CHOP, and the effects of CQ use. The elucidation of ERS and UPR in the exocrine glands of SD patients may contribute to a better understanding of the cell membrane disorder and the protein secretory pathway, and the identification of new targets for therapeutic interventions.

## Methods

### Study design and population

A cross-sectional study was conducted at the University Hospital of Ribeirão Preto Medical School – University of São Paulo, Brazil. Consecutive patients admitted to the outpatient clinic for diagnostic evaluation of SD were studied. Subjects were classified according to the 2016 American College of Rheumatology and European League Against Rheumatism (ACR-EULAR) criteria as pSD, aSD, or NSS [2]. The HC group consisted of volunteers with no dryness and no history of AID, who underwent oral surgery for benign noninflammatory oral diseases involving the lower lip. All patients underwent a clinical evaluation and the unstimulated salivary flow test, whose results above 0.1 mL/min were considered normal. Four groups of patients were constituted: pSD, aSD, NSS, and HC. The pSD and aSD groups were combined in a single SD group for further comparisons. The expression of ERS was also compared between patients using CQ (CQ group) and those not using CQ (non-CQ group). Healthy volunteers were not included in the non-CQ group.

### Biopsies

A labial salivary gland biopsy was performed as previously described [22]. The specimens included at least six or more glands; four samples were conventionally fixed for histopathological analysis (Hematoxylin & Eosin), and at least two samples were used for mRNA extraction. The samples for mRNA extraction were immediately frozen and stored at -80 °C.

### Serological evaluation

All patients with suspected SD were tested for anti-Ro/SSA antibodies, whereas asymptomatic controls with no history of AID were not tested. Commercially available SS-A Elisa Kits from QUANTA Lite^®^ (Inova Diagnostics, San Diego, USA) were used, following the manufacturer’s protocol to measure Ro antibodies with the BioTek ELx808^®^ reader. Data were analyzed using the Gen5^®^ software, and the samples were considered positive for values above 20, according to the specifications of the kit.

### ERS-related genes

The genes selected for analysis of ERS were BIP, PERK, XBP1, ATF-6, ATF-4, and CHOP. These genes participate in the three major UPR pathways and apoptosis [5]. Both XBP1 and ATF-6 regulate the expression of various genes involved in ERAD [10]. CALR and CANX were also analyzed because both are calcium-dependent and intracellular calcium depletion present in RAD may affect ERS balance [12, 13]. The constitutive gene glyceraldehyde-3-phosphate dehydrogenase (GAPDH) was used as an endogenous control. The primer sequences used are presented in supplementary Table 1.

### RNA extraction

For RNA extraction, the TRIzol Sigma TRI-Reagent^®^ was used. Briefly, after total trituration of the tissue sample in the presence of TRIzol, 1/5 (v/v) chloroform was added to the tube. The samples were left to stand at room temperature for 10 min and then centrifuged in an Eppendorf centrifuge at 12,000 × g for 15 min at 4 °C. After centrifugation, the colorless phase of the tube was transferred to a new tube for RNA precipitation in the presence of 1:1 isopropyl alcohol and centrifuged again at 12,000 × g for 10 min at 4 °C. A precipitate containing RNA was identified and washed with 75% ethanol; the ethanol was then removed, the precipitate was dried, and RNase-free water was added. RNA was quantified with a SpectraMax M3 reader from Molecular Devices^®^.

### cDNA synthesis

For cDNA synthesis, 1000 ng of µRNA was treated with 1 µL of DNAse + 1 µL of buffer for a total volume of 10 µL and incubated at 37 °C for 30 min. At the end of the reaction, 1 µl of STOP solution was added, and the mixture was maintained at 65 °C for 10 min. The reverse transcription technique was applied for cDNA production using 11 µL of DNAse-treated RNA, 4 µL of Buffer 5x iScript™ Reaction Mix^®^ (BIO-RAD^®^, Hercules, USA), 1 µL of iScript™ Reverse Transcriptase^®^ (BIO-RAD^®^, Hercules, USA), and 4 µL of RNAse-free water. The samples were processed with a Thermal Cycler C1000 Touch™ CFX96 Real-Time System^®^ under the following conditions: at 25 °C for 5 min, at 42 °C for 30 min, at 85 °C for 5 min and at 12 °C thereafter. Finally, 180 µL of RNAse-free water was added to the samples to a final RNA concentration of 5 ng/mL.

### qPCR analysis

For the qPCR procedure, 2 µL of cDNA was transferred to qPCR microplates, followed by the addition of 5 µL of EvaGreen^®^ reagent (BIO-RAD^®^, Hercules, USA), 0.3 µL of forward primer (1:10 dilution), 0.3 µL of reverse primer (1:10 dilution) and 2.4 µL of RNase-free water, for a total solution volume of 10 µL. The sequence of primer oligonucleotides was acquired from Sigma-Aldrich^®^, Darmstadt, Germany (Supplementary Table 1). A positive control for gene expression, a blank control (water) and a no template control (NTC) were added to each plate. To determine gene expression, the samples were subjected to a CFX96 Touch™ Real-Time PCR Detection System under the following conditions: 40 cycles at 95 °C for 35 s, and at 60 °C for 30 s. Dissociation protocols were used to test the efficacy of the primers for specific gene amplification. All experiments were performed in duplicate.

### Statistical analysis

Data were presented as means and standard errors or medians and interquartile ranges, as appropriate. The Shapiro-Wilk and Levene tests were performed to evaluate normality and homogeneity of variance. Between-group comparisons were performed using the Kruskal–Wallis test with post-hoc Dunn’s multiple comparison test and the Mann–Whitney test. Statistical significance was set at *p*-value < 0.05. The analysis was performed using Software R (version 4.3.1, R Core Team, Vienna, Austria). Data are presented in tables, box plots, and supplementary tables.

## Results

A total of 57 female patients participated in the study. Forty-four patients aged 18 to 75 years with dryness symptoms were classified as having SD or NSS. Sixteen patients had pSD, 12 had aSD, and 16 had NSS. The HC group consisted of 13 individuals without dryness symptoms.

Three patients who exhibited clinical features similar to SD at first, but met exclusion criteria according to the 2016 ACR-EULAR consensus (one patient had hepatitis C, one patient had IgG4-related disease, and one patient had graft-versus-host disease) were subsequently classified as NSS. In two healthy volunteers, a low unstimulated salivary flow rate was detected, but histopathological examination showed normal salivary glands. A detailed description of demographic characteristics, focus score, salivary flow rate, and autoantibody data of the pSD, aSD, NSS, and HC groups is presented in Table 1. The CQ group comprised 16 patients and non-CQ group comprised 29 patients.

**Table 1:** Demographic characteristics, focus score, salivary flow, and serological results of the four groups

	pSD	aSD	NSS	HC
N	16	12	16	13
Gender N (female/male)	16/0	12/0	16/0	13/0
Mean age ± S.D. (years)	44,0 ± 14,5	51,5 ± 12,6	48,7 ± 13,7	43,3 ± 14,2
Focus score^a^				
0	1	3	12	11
1	4	3	4*	2
2	3	0	0	0
3	1	1	0	0
4	7	5	0	0
UWSF				
< 0.1 mL/min	6	7	10	2
≥ 0.1 mL/min	10	5	6	11
Positive Ro antibodies	13	9	2*	NT

pSD, Primary Sjögren disease; aSD, Associated Sjögren disease; NSS, Non-Sjögren’s Sicca; HC, Healthy control; N, Number; UWSF, Unstimulated whole salivary flow; S.D., Standard deviation; NT: Not tested

a: Number of foci per 4 mm^2^ of tissue

*Patients with other diseases that meet the exclusion criteria, according to the 2016 ACR/EULAR consensus (hepatitis C and IgG4-related disease)

### Expression of genes involved in ERS

**Fig. 2:**
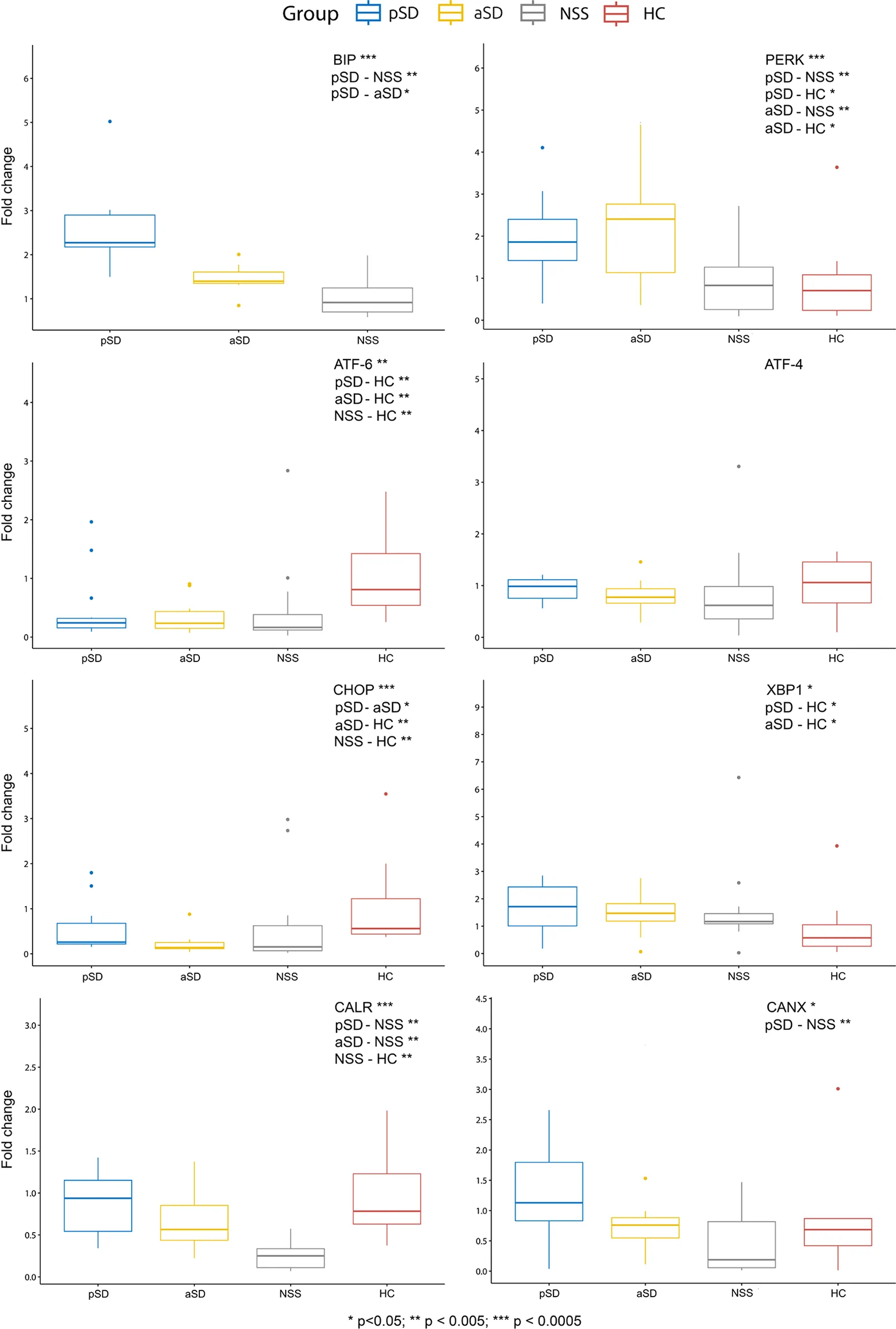
Distribution of ERS genes among the four groups (pSD, aSD, NSS, and HC). *Legend 2* The expression of BIP is notably higher in the pSD group compared to other groups, and it is undetectable in the HC group. PERK exhibits significant upregulation in both pSD and aSD groups compared to the NSS and HC groups. In contrast, ATF-6 was downregulated in the pSD, aSD, and NSS groups compared to the HC group. There was no significant difference in ATF-4 expression between groups. CHOP expression was lower among patients than healthy controls, mainly in the aSD group. There was an overexpression of XBP1 in both pSD and aSD groups compared to the HC group. CALR expression was significantly lower in the NSS group. Finally, CANX expression was significantly higher in the SD groups (pSD and aSD) compared to the NSS and HC groups

**Fig. 3:**
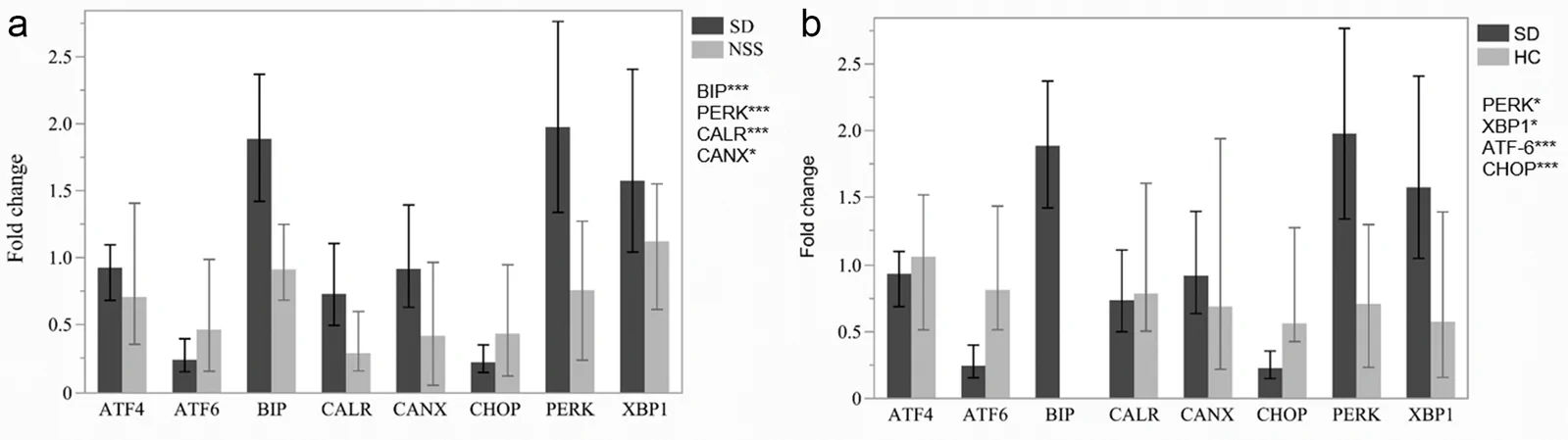
Expression of ERS genes in the SD group compared to the NSS and HC groups. *Legend 3* Graph **A** Patients with SD showed significantly higher expression of BIP, PERK, CALR, and CANX than NSS patients. Graph **B** Patients with SD showed significantly higher expression of PERK and XBP1, and lower expression of ATF-6 and CHOP compared with healthy controls (HC). BIP expression was undetectable in the HC group; the error bars represent the upper and lower quartiles. Significance levels are represented as *p < 0.05; **p < 0.01; ***p < 0.005

Results of mRNA expression in the labial salivary glands of patients and healthy volunteers are described in Fig. 2 and Supplementary Table 2. The groups of patients with pSD and aSD were merged into the SD group, which was also compared to the NSS and HC groups (Fig. 3 and Supplementary Table 3). Expressions of BIP (*p* < 0.0001), PERK (*p* = 0.0027), ATF-6 (*p* = 0.0013), CHOP (*p* = 0.0007), CALR (*p* = 0.0002), and CANX (*p* = 0.0283) mRNA were statistically different, while ATF-4 (*p* = 0.308) and XBP1 (*p* = 0.149) expressions were not different between the four groups.

There was significantly higher expression of BIP in patients with SD compared to NSS (*p* < 0.0001); the expression was higher in the pSD group compared to the aSD (*p* = 0.0125) and the NSS (*p* < 0.0001) groups. BIP was undetectable in the HC group. There was no statistical difference between the aSD and the NSS groups.

The expression of PERK was higher in patients with SD than in individuals with NSS (*p* = 0.0003) or HC (*p* = 0.0117). The differences were also significant when pSD was compared to NSS (*p* = 0.0027) and HC (*p* = 0.0107), and when aSD was compared to NSS (*p* = 0.0091) and HC (*p* = 0.0232). No difference was observed between pSD and aSD.

The expression of ATF-6 was downregulated in pSD, aSD, and NSS compared to the HC group (*p* = 0.0030, *p* = 0.0029, *p* = 0.0002 respectively). Expression of ATF-6 was also significantly lower in the SD group (pSD + aSD) as compared with HC (*p* = 0.0003), but not with the NSS group. ATF-4 expression showed no significant difference in any of the comparative analyses.

The expression of CHOP was lower in SD patients than in HC (*p* = 0.0003). Comparative analysis revealed significantly lower expressions of CHOP in aSD versus pSD (*p* = 0.0490), in aSD versus HC (*p* = 0.0002); and in NSS versus HC (*p* = 0.0008). Regarding XBP1 expression, a higher expression was observed in pSD versus HC (*p* = 0.0374) and aSD versus HC (*p* = 0.0397), resulting in higher expression in the SD versus the HC group (*p* = 0.0346).

The expression of CALR was lower in NSS patients in all comparisons, i.e., pSD versus NSS (*p* < 0.0001), aSD versus NSS (*p* = 0.0087), and HC versus NSS (*p* = 0.0018). Group analysis revealed no significant difference between the SD and HC groups, although CALR expression was significantly lower in NSS than in SD (*p* < 0.0001). In contrast, CANX expression was upregulated, especially in pSD, but the difference was significant only between pSD and NSS (*p* = 0.0031). However, CANX expression was significantly higher in SD than in NSS (*p* = 0.0111).

**Fig. 4:**
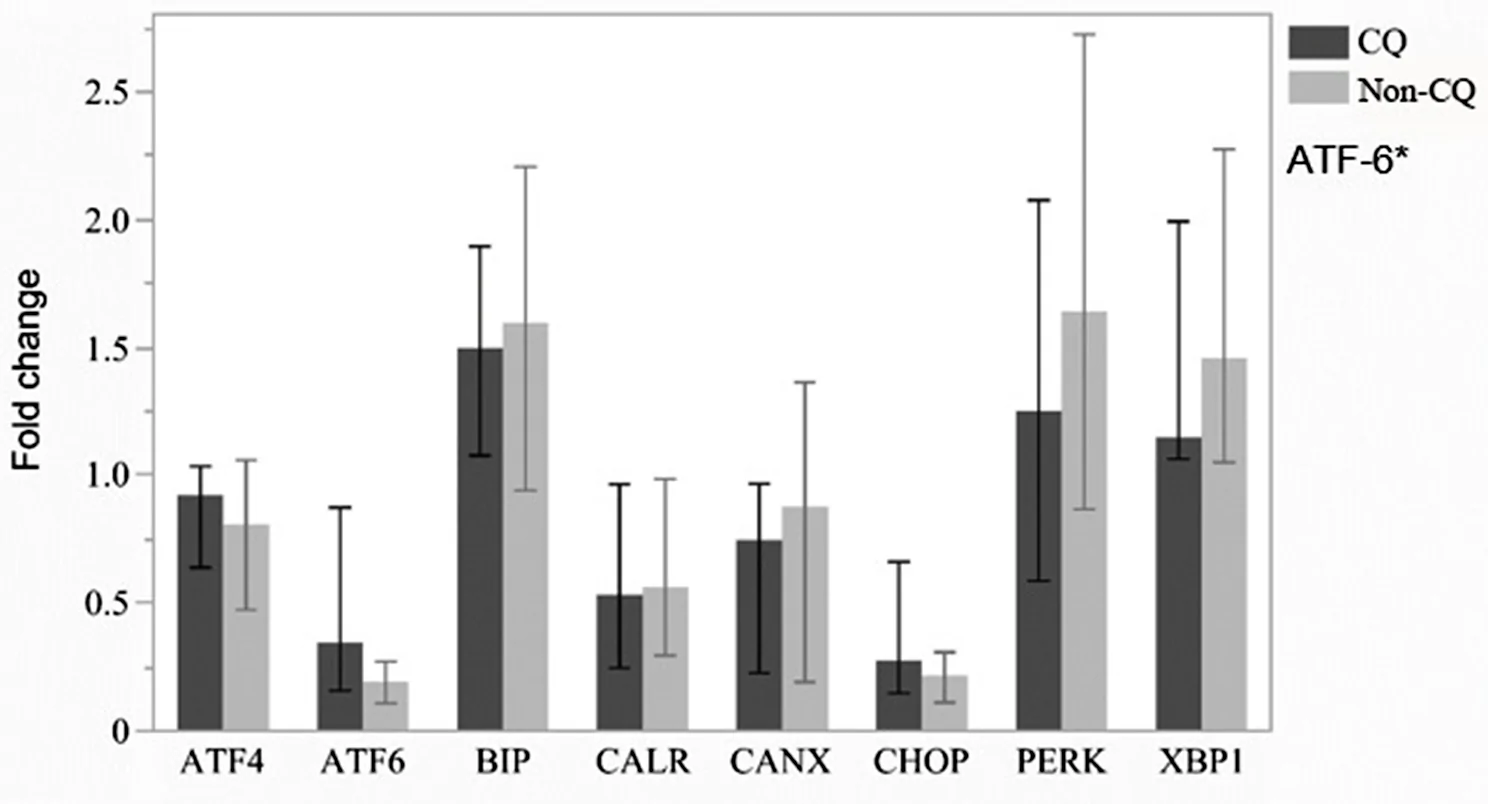
Expression of ERS genes in the SD group undergoing treatment with chloroquine or not. *Legend 4* Comparison of the expression of ERS-related genes between Sjögren’s disease patients taking chloroquine (CQ) or not (non-CQ); there was a significantly higher expression of ATF-6 in the group treated with CQ; the error bars represent the upper and lower quartiles. Significance levels are denoted as follows: *p < 0.05

Concerning the effect of CQ on ERS expression, only ATF-6 was significantly upregulated in the CQ group (*p* = 0.0317) (Fig. 4 and Supplementary Table 4).

## Discussion

Abnormal ERS is closely related to AID. The orchestration of this process may vary depending on the type of cell involved, culminating in the activation/inactivation of different genes and inflammatory pathways. ERS is involved, for example, in the B cells’ differentiation into plasma cells and increased antibody secretion, the activation of toll-like receptors and the release of proinflammatory cytokines by macrophages, antigen presentation, T cell apoptosis, and even in the production and release of neutrophil extracellular traps. Apart from the immune system cells, the resident cells of target tissues are also affected [23]. This sort of dysfunction has been documented in lupus nephritis, where some activated pathways (IRE1α-XBP1) have a protective role for renal podocytes, while others (PERK-ATF4-CHOP) induce their apoptosis, as well as that of tubular epithelial cells [24].

In ERS conditions, increased BIP levels are expected, since the accumulation of unfolded proteins triggers the dissociation of BIP from PERK, IRE1α, and ATF-6 [25]. In the present study, we found an upregulation of BIP expression in salivary gland cells of SD patients, particularly in pSD (as compared with aSD) patients, suggesting higher ERS in the former group. The undetectable amount of BIP in our HC group confirms the quality of the assays since they were performed in duplicate on the same plate, with the same batch of primer as the others. The few studies available on ERS in SD patients did not describe in detail the profile of their controls [20, 21, 26]. In addition, the expression of BIP in NSS subjects suggests that other conditions, not necessarily those of an autoimmune nature, may be associated with ERS in these patients. Endogenous or exogenous factors, such as infection, calcium imbalance, and drug toxicity may account for the misfolded protein imbalance in these cases. Although more comprehensive information is necessary, BIP could serve as a potential marker in SD investigations.

In agreement, PERK, one of the main UPR pathways, was the second most expressed gene. After BIP dissociation, the release and activation of PERK lead to eIF2α phosphorylation, which temporarily attenuates protein translation and acts on ATF-4 to increase the transcription of proteins that promote cell survival under ERS conditions [6]. The significant increase in PERK in pSD and aSD, as in the SD group, compared to NSS and HC groups reaffirms ERS in the salivary tissue of patients with SD. The increased expression of PERK, however, was not accompanied by an increase in ATF-4. In this study, ATF-4 expression is similar in all groups, suggesting that something interrupts the UPR pathway progression and may perpetuate a chronic ERS status. Inversely to our results, Sepulveda et al.. have previously described BIP reduction associated with IRE1α and XBP1 reduction in SD patients [21], while Bahamondes et al. detected increased expression of ATF-4 in patients with SD, although PERK activation was similar to that in controls [26].

The ATF-6 is a type II transmembrane protein of the ER that is activated in ERS and translocated to the Golgi complex, where it is cleaved by S1P and S2P proteases, releasing ATF-6 (N) fragments with a free N-terminal portion into the cytosol. ATF-6 (N) migrates to the nucleus and activates the transcription of UPR and ERAD genes. The ERAD system is responsible for the removal of unfolded proteins from the ER lumen and their transport to the cytoplasm so that they may be degraded, facilitating homeostasis [5]. Takahata et al. reported that the 52-kDa isoform of the SSA/Ro protein (Ro52) has E3 ubiquitin ligase activity. The protein E3 ubiquitin is part of the ERAD system that promotes the degradation of unfolded IgG1 immunoglobulins [27]. In a study of 13 patients with pSD, Barrera et al.. detected an increase in ATF-6 expression and the ERAD pathway associated with proinflammatory cytokines [20]. In the current investigation, however, we detected a reduction of ATF-6 in SD, as in both pSD and aSD groups, compared to HC. It is impossible to state that ERAD is downregulated since other genes or proteins were not analyzed. Still, this study suggests this UPR pathway is not upregulated by ATF-6, unlike previous studies. Although the ATF-6 expression was not different between the NSS and the SD groups, the significantly lower expression of ATF-6 in NSS subjects when compared to the HC group suggests that clinical changes may lead to alterations in the ERS in these patients.

Another relevant aspect is that PERK hyperactivity accelerates cell death by CHOP upregulation and inhibition of the antiapoptotic gene BCL-2 [5]. The process of apoptosis leading to dysfunction of the exocrine glands and reduced salivary and tear flow is previously demonstrated in SD patients [28, 29]. Herein, despite the increase in PERK, ATF-4 was not different between groups and CHOP was significantly lower in the SD group, especially in aSD compared to pSD and HC. The reduction of ATF-6 and CHOP in SD compared to the HC group suggests a chronic ERS status and reduction in apoptosis, which may increase the risk of lymphoma in SD patients. In accordance, Manganelli and Fietta reported a correlation of BLC-2 expression with prolongation of the inflammatory process and the risk of developing lymphoma in SD patients [28]. There was lower expression of CHOP and ATF-6 in NSS compared to HC, reaffirming that clinical changes in NSS patients may also interfere with this pathway.

The IRE1α removes the 26-nt intron from XBP1 mRNA, fragmenting it into two mRNA segments that are unified by a ligase capable of producing the transcription factor XBP1s (the spliced form of XBP1), which in turn contains an activated domain that permits its translocation to the nucleus. This process induces the transcription of various genes that increase the size and function of the ER, promoting better protein transport and degradation of unfolded proteins [4]. While Wang et al. detected positive regulation of XBP1 in 76 patients with SLE [30], Sepulveda et al. detected a reduction of XBP1 in 47 patients with SD [21]. In the present study, when pSD, aSD, and SD were compared to the HC group, XBP1 was meaningly higher, suggesting activation of ERS in SD patients.

CALR and CANX are calcium-dependent ER proteins with a high degree of similarity that act in conjunction under ERS conditions [6, 31]. The pro-inflammatory state of RAD is known to promote intracellular calcium depletion [18], a UPR activator. Staikou et al. demonstrated the association of anti-Ro with CALR in serum samples of patients with SLE and SD [32]. Weber et al.. detected anti-CANX antibodies in patients with SLE and rheumatoid arthritis [33]. Although there is no study of CANX in SD to date, in our study the NSS group showed significantly lower expression of CANX as compared with pSD but not with aSD, suggesting higher ERS expression in pSD. CALR was less expressed in the NSS group compared to pSD, aSD, SD, and HC groups. No previous study analyzed CALR in specimens of salivary glands, only in serum samples. Thus, this is a new finding about CALR that requires further investigation.

The CQ was the first potent and mass-producible drug against malaria [34]. The first positive effects of CQ on RADs were observed during World War II; soldiers taking CQ as prophylaxis reported improvement in rashes and inflammatory arthritis [35]. Today, CQ is commonly used to treat rheumatic and dermatological diseases due to its effects on at least four sets of cellular functions – endolysosomal activities, including autophagy; cytokine signaling, including Toll-like receptor; NADPH oxidase signaling; and calcium mobilization from the ER [35]. These effects might further modulate other cellular and organismal processes, such as ER-to-Golgi trafficking, although the underlying mechanisms remain to be identified [36]. Tian et al. observed in vitro (human cultured cells) and in vivo (mice) effects of CQ over ERS, inducing phosphorylation of eIF2α, activation of CHOP, upregulation of ATF-6, and activation of XBP1 [37]. However, in quantitative terms, the effects of CQ on these transcription factors appear relatively minor when compared to positive controls to elicit ERS [37]. Although we did not observe an effect of CQ on all UPR pathways, our findings corroborate an upregulation of at least ATF-6 in SD patients treated with CQ. The discrepancies observed between the studies may be attributable to the influence of other pharmacological agents and coexisting medical conditions on the pathways involved in ERS. Despite that, this study suggests that CQ acts on one of the mechanisms underlying the ERS, with this pathway being a possible target for treating SD patients.

Overall, this study identified distinct patterns in stress response between patients with SD and NSS, revealing that the NSS group exhibited responses more closely aligned with SD than with HC in the context of the ERS. In SD patients, ERS appears to adopt a chronic course, as indicated by heightened expression of both PERK and BIP and reduced apoptotic signaling, evidenced by low levels of ATF-6 and CHOP. Notably, CQ may be protective by preventing apoptosis while maintaining elevated levels of ATF-6 in patients receiving CQ compared to those not.

The main limitation of this study is the lack of assessment of correlations of RNA expression data with protein expression as well as functional changes in apoptosis or autophagy. Furthermore, the number of patients in each group did not allow the analysis of relevant data, such as correlations with the sub-phenotypes of SD (pure glandular or systemic manifestations), with the sort of overlap in aSD, nor with pharmacological regimens or disease activity. Despite these limitations, this study is the first to demonstrate an association between SD and ERS with marked differences in a well-characterized group of patients. This new information may help guide future studies in identifying therapeutic targets for SD patients.

## Conclusion

This study explores the activity of endoplasmic reticulum stress (ERS) in minor salivary gland tissues, highlighting varying degrees of involvement among patients with primary Sjögren’s syndrome (pSD), associated Sjögren’s syndrome (aSD), and non-Sjögren’s sicca (NSS). The observed differences in mRNA expression raise questions about whether these variations reflect disease-specific characteristics or the influence of therapeutic regimens to which individuals were exposed. Further research is needed to elucidate how molecular chaperones, autophagy, and proteasome degradation pathways mitigate ERS, restore ER homeostasis, and potentially improve immune cell dysfunction and resident cell damage.

## Electronic supplementary material

Below is the link to the electronic supplementary material.

Supplementary Material 1

Supplementary Material 2

Supplementary Material 3

Supplementary Material 4

## Data Availability

No datasets were generated or analysed during the current study.

## Abbreviations

ACR-EULAR: American College of Rheumatology /European League Against Rheumatism
AID: Autoimmune diseases
aSD: Associated Sjögren’s disease
ATF-4: Activating transcription factor 4
ATF-6: Activating transcription factor 6
BCL-2: B-cell lymphoma 2 protein
BIP: Binding protein, also known as GRP78
CALR: Calreticulin
CANX: Calnexin
CAPES: Coordenação de Aperfeiçoamento de Pessoal de Nível Superior
CHOP: C/EBP- homologous protein ou GADD153
CQ: Chloroquine
DM: Diabetes mellitus
eIF2α: Eukaryotic translation Initiation Factor 2α
ER: Endoplasmic reticulum
ERAD: ER-associated protein degradation
ERp57: Endoplasmic reticulum–resident protein 57
ERS: Endoplasmic reticulum stress
GAPDH: Glyceraldehyde-3-phosphate dehydrogenase
HC: Healthy control
IRE1α: Inositol-requiring enzyme 1α
IFN: Interferon
NSS: Non-Sjögren sicca
PERK: Protein endoplasmic reticulum kinase
pSD: Primary Sjögren’s disease
RAD: Rheumatic autoimmune diseases
SD: Sjögren’s disease
SLE: Systemic lupus erythematosus
UPR: Unfolded protein response
XBP1: x-box protein 1
