# Correction: Endoplasmic reticulum stress in the salivary glands of patients with primary and associated Sjögren’s disease, and non-Sjögren’s sicca syndrome: a comparative analysis and the influence of chloroquine

**DOI:** 10.1186/s42358-025-00436-9

**Published:** 2025-01-21

**Authors:** Graziela Vieira Cavalcanti, Fabiola Reis de Oliveira, Rafael Ferraz Bannitz, Natalia Aparecida de Paula, Ana Carolina Fragoso Motta, Eduardo Melani Rocha, John Chiorini, Hilton Marcos Alves Ricz, Denny Marcos Garcia, Maria Cristina Foss-Freitas, Luiz Carlos Conti de Freitas

**Affiliations:** 1 Department of Ophthalmology, Otolaryngology, Head and Neck Surgery, Ribeirão Preto Medical School, University of São Paulo, São Paulo, Brazil; 2 Ophthalmology, Otolaryngology, Head and Neck Surgery Department, Ribeirão Preto Medical School, University of São Paulo, Av. Bandeirantes 3900, Monte Alegre, Ribeirão Preto, SP 14.040-900 Brazil; 3 Department of Internal Medicine, Ribeirão Preto Medical School, University of São Paulo, São Paulo, Brazil; 4 Department of Stomatology, Public Health and Forensic Dentistry, Ribeirão Preto School of Dentistry, University of São Paulo, São Paulo, Brazil; 5 Adeno-Associated Virus Biology Section, National Institute of Dental and Craniofacial Research, National Institutes of Health, Bethesda, MD USA; 6 Division of Metabolism, Endocrinology, and Diabetes, Department of Internal Medicine, and Caswell Diabetes Institute, University of Michigan, Ann Arbor, MI USA

**Correction: Advances in Rheumatology (2024) 65:2**

10.1186/s42358-024-00430-7

The original publication of this article contained an incorrect title. The incorrect and correct title are listed in this correction article. The original article has been updated.

Incorrect

Endoplasmic reticulum stress in the salivary glands of patients with primary Sjögren’s syndrome, associated Sjögren’s syndrome, and non-Sjögren’s sicca syndrome: a comparative analysis and the influence of chloroquine

Correct

Endoplasmic reticulum stress in the salivary glands of patients with primary and associated Sjögren’s disease, and non-Sjögren’s sicca syndrome: a comparative analysis and the influence of chloroquine

