# Supplementary material for: Endoplasmic reticulum stress in the salivary glands of patients with primary and associated Sjögren’s disease, and non-Sjögren’s sicca syndrome: a comparative analysis and the influence of chloroquine
Source: Adv Rheumatol. 2025 Jan 8;65:2. doi: 10.1186/s42358-024-00430-7 (PMC13488755; doi:10.1186/s42358-024-00430-7)
Supplement: Supplementary file 1 [file 42358_2024_430_MOESM1_ESM.docx]

**Supplementary table 1**

Sequence of oligonucleotides from Sigma-Aldrich® (Merck, Darmstadt, Germany) used to analyze ERE expression.

| **Gene** | **Forward** | **Reverse** |
| --- | --- | --- |
| BIP | CATCACGCCGTCCTATGTCG | CGTCAAAGACCGTGTTCTCG |
| PERK | TGTCGCCAATGGGATAGTGACGAA | AATCCGGCTCTCGTTTCCATGTCT |
| XBP1 | TGGCCGGGTCTGCTGAGTCCG | ATCCATGGGAAGATGTTCTGG |
| ATF-6 | AAGGCTTGGGCTGAATTGAA | ATGTCTCCCCTTTCCTTATATGGT |
| ATF-4 | GGGTTCTCCAGCGACAAGGCTAAG | AACAGGGCATCCAAGTCGAACTC |
| CALR | CCTGCCGTCTACTTCAAGGAG | GAACTTGCCGGAACTGAGAAC |
| CANX | CCAAGGTTACTTACAAAGCTCCA | GGCCCGAGACATCAACACA |
| CHOP | GGAGAACCAGGAAACGGAAAC | TCTCCTTCATGCGCTGCTTT |
| GAPDH | AGGGCTGCTTTTAACTCTGGT | CCCCACTTGATTTTGGAGGGA |
