# Supplementary material for: Endoplasmic reticulum stress in the salivary glands of patients with primary and associated Sjögren’s disease, and non-Sjögren’s sicca syndrome: a comparative analysis and the influence of chloroquine
Source: Adv Rheumatol. 2025 Jan 8;65:2. doi: 10.1186/s42358-024-00430-7 (PMC13488755; doi:10.1186/s42358-024-00430-7)
Supplement: Supplementary file 2 [file 42358_2024_430_MOESM2_ESM.docx]

**Supplementary Table 2**

Statistical findings in the four groups (pSD, aSD, NSS, and HC).

|  | **Group** | **N** | **Median** | **IQR** | | **Mean** | **IC 95%** | |
| --- | --- | --- | --- | --- | --- | --- | --- | --- |
|  |  |  |  | **P25** | **P75** |  | **Lower** | **Upper** |
| ATF4 | HC | 6 | 1.06 | 0.51 | 1.52 | 1.00 | 0.36 | 1.64 |
|  | NSS | 13 | 0.62 | 0.35 | 1.00 | 0.86 | 0.35 | 1.37 |
|  | pSD | 13 | 0.99 | 0.74 | 1.13 | 0.93 | 0.81 | 1.06 |
|  | aSD | 10 | 0.78 | 0.64 | 0.98 | 0.82 | 0.59 | 1.05 |
| ATF6 | HC | 13 | 0.81 | 0.51 | 1.44 | 1.00 | 0.62 | 1.38 |
|  | NSS | 16 | 0.17 | 0.10 | 0.39 | 0.43 | 0.06 | 0.80 |
|  | pSD | 16 | 0.24 | 0.15 | 0.33 | 0.43 | 0.15 | 0.71 |
|  | aSD | 12 | 0.24 | 0.12 | 0.47 | 0.34 | 0.15 | 0.52 |
| BIP | HC | 0 | - | - | - | - | - | - |
|  | NSS | 11 | 0.92 | 0.69 | 1.25 | 1.03 | 0.73 | 1.32 |
|  | pSD | 11 | 2.27 | 2.16 | 2.91 | 2.56 | 1.92 | 3.20 |
|  | aSD | 9 | 1.40 | 1.33 | 1.69 | 1.47 | 1.22 | 1.71 |
| CALR | HC | 5 | 0.78 | 0.50 | 1.61 | 1.00 | 0.22 | 1.78 |
|  | NSS | 12 | 0.25 | 0.11 | 0.42 | 0.27 | 0.16 | 0.38 |
|  | pSD | 15 | 0.94 | 0.52 | 1.17 | 0.88 | 0.68 | 1.08 |
|  | aSD | 11 | 0.57 | 0.35 | 0.96 | 0.66 | 0.43 | 0.89 |
| CANX | HC | 5 | 0.69 | 0.22 | 1.94 | 1.00 | -0.45 | 2.45 |
|  | NSS | 12 | 0.19 | 0.05 | 0.98 | 0.46 | 0.14 | 0.78 |
|  | pSD | 15 | 1.13 | 0.72 | 2.11 | 1.31 | 0.89 | 1.73 |
|  | aSD | 11 | 0.76 | 0.53 | 0.90 | 0.73 | 0.46 | 0.99 |
| CHOP | HC | 13 | 0.56 | 0.42 | 1.27 | 1.00 | 0.45 | 1.55 |
|  | NSS | 16 | 0.15 | 0.05 | 0.65 | 0.57 | 0.07 | 1.06 |
|  | pSD | 16 | 0.26 | 0.21 | 0.71 | 0.51 | 0.24 | 0.77 |
|  | aSD | 12 | 0.14 | 0.11 | 0.29 | 0.22 | 0.08 | 0.36 |
| PERK | HC | 8 | 0.71 | 0.23 | 1.30 | 1.00 | 0.04 | 1.96 |
|  | NSS | 14 | 0.83 | 0.25 | 1.27 | 0.92 | 0.47 | 1.38 |
|  | pSD | 14 | 1.86 | 1.39 | 2.65 | 2.01 | 1.48 | 2.55 |
|  | aSD | 10 | 2.41 | 0.94 | 2.86 | 2.14 | 1.18 | 3.09 |
| XBP1 | HC | 8 | 0.57 | 0.16 | 1.39 | 1.00 | -0.07 | 2.07 |
|  | NSS | 15 | 1.17 | 1.09 | 1.55 | 1.56 | 0.76 | 2.36 |
|  | pSD | 14 | 1.71 | 0.98 | 2.47 | 1.67 | 1.16 | 2.18 |
|  | aSD | 12 | 1.47 | 1.16 | 1.94 | 1.50 | 1.02 | 1.99 |

(*) Kruskal-Wallis test; IQR = interquartile range; IC 95% = confidence interval 95%
