# Supplementary material for: Endoplasmic reticulum stress in the salivary glands of patients with primary and associated Sjögren’s disease, and non-Sjögren’s sicca syndrome: a comparative analysis and the influence of chloroquine
Source: Adv Rheumatol. 2025 Jan 8;65:2. doi: 10.1186/s42358-024-00430-7 (PMC13488755; doi:10.1186/s42358-024-00430-7)
Supplement: Supplementary file 3 [file 42358_2024_430_MOESM3_ESM.docx]

**Supplementary Table 3**

Statistical findings in the SD group compared to the NSS group.

|  | **Group** | **N** | **Median** | **IQR** | | **Mean** | **IC 95%** | |
| --- | --- | --- | --- | --- | --- | --- | --- | --- |
|  |  |  |  | **P25** | **P75** |  | **Lower** | **Upper** |
| ATF4 | NSS | 13 | 0.62 | 0.35 | 1.00 | 0.86 | 0.35 | 1.37 |
|  | SD | 23 | 0.93 | 0.69 | 1.10 | 0.88 | 0.77 | 1.00 |
| ATF6 | NSS | 16 | 0.17 | 0.10 | 0.39 | 0.43 | 0.06 | 0.80 |
|  | SD | 28 | 0.24 | 0.15 | 0.40 | 0.39 | 0.22 | 0.56 |
| BIP | NSS | 11 | 0.92 | 0.69 | 1.25 | 1.03 | 0.73 | 1.32 |
|  | SD | 20 | 1.89 | 1.42 | 2.37 | 2.07 | 1.64 | 2.50 |
| CALR | NSS | 12 | 0.25 | 0.11 | 0.42 | 0.27 | 0.16 | 0.38 |
|  | SD | 26 | 0.73 | 0.50 | 1.11 | 0.79 | 0.64 | 0.94 |
| CANX | NSS | 12 | 0.19 | 0.05 | 0.98 | 0.46 | 0.14 | 0.78 |
|  | SD | 26 | 0.92 | 0.63 | 1.40 | 1.06 | 0.78 | 1.34 |
| CHOP | NSS | 16 | 0.15 | 0.05 | 0.65 | 0.57 | 0.07 | 1.06 |
|  | SD | 28 | 0.22 | 0.15 | 0.35 | 0.39 | 0.22 | 0.55 |
| PERK | NSS | 14 | 0.83 | 0.25 | 1.27 | 0.92 | 0.47 | 1.38 |
|  | SD | 24 | 1.98 | 1.34 | 2.76 | 2.07 | 1.60 | 2.53 |
| XBP1 | NSS | 15 | 1.17 | 1.09 | 1.55 | 1.56 | 0.76 | 2.36 |
|  | SD | 26 | 1.58 | 1.05 | 2.41 | 1.59 | 1.26 | 1.92 |

(*) Mann-Whitney test; IQR = interquartile range; IC 95% = confidence interval 95%

Statistical findings in the SD group compared to the HC group.

|  | **Group** | **N** | **Median** | **IQR** | | **Mean** | **IC 95%** | |
| --- | --- | --- | --- | --- | --- | --- | --- | --- |
|  |  |  |  | **P25** | **P75** |  | **Lower** | **Upper** |
| ATF4 | HC | 6 | 1.06 | 0.51 | 1.52 | 1.00 | 0.36 | 1.64 |
|  | SD | 23 | 0.93 | 0.69 | 1.10 | 0.88 | 0.77 | 1.00 |
| ATF6 | HC | 13 | 0.81 | 0.51 | 1.44 | 1.00 | 0.62 | 1.38 |
|  | SD | 28 | 0.24 | 0.15 | 0.40 | 0.39 | 0.22 | 0.56 |
| BIP | HC | 0 | -. | - | - | - |  |  |
|  | SD | 20 | 1.89 | 1.42 | 2.37 | 2.07 | 1.64 | 2.50 |
| CALR | HC | 5 | 0.78 | 0.50 | 1.61 | 1.00 | 0.22 | 1.78 |
|  | SD | 26 | 0.73 | 0.50 | 1.11 | 0.79 | 0.64 | 0.94 |
| CANX | HC | 5 | 0.69 | 0.22 | 1.94 | 1.00 | -0.45 | 2.45 |
|  | SD | 26 | 0.92 | 0.63 | 1.40 | 1.06 | 0.78 | 1.34 |
| CHOP | HC | 13 | 0.56 | 0.42 | 1.27 | 1.00 | 0.45 | 1.55 |
|  | SD | 28 | 0.22 | 0.15 | 0.35 | 0.39 | 0.22 | 0.55 |
| PERK | HC | 8 | 0.71 | 0.23 | 1.30 | 1.00 | 0.04 | 1.96 |
|  | SD | 24 | 1.98 | 1.34 | 2.76 | 2.07 | 1.60 | 2.53 |
| XBP1 | HC | 8 | 0.57 | 0.16 | 1.39 | 1.00 | -0.07 | 2.07 |
|  | SD | 26 | 1.58 | 1.05 | 2.41 | 1.59 | 1.26 | 1.92 |

(*) Mann-Whitney test; IQR = interquartile range; IC 95% = confidence interval 95%
