# Supplementary material for: Endoplasmic reticulum stress in the salivary glands of patients with primary and associated Sjögren’s disease, and non-Sjögren’s sicca syndrome: a comparative analysis and the influence of chloroquine
Source: Adv Rheumatol. 2025 Jan 8;65:2. doi: 10.1186/s42358-024-00430-7 (PMC13488755; doi:10.1186/s42358-024-00430-7)
Supplement: Supplementary file 4 [file 42358_2024_430_MOESM4_ESM.docx]

**Supplementary Table 4**

Statistical findings in the CQ group compared to the Not CQ group.

|  | **Group** | **N** | **Median** | **IQR** | | **Mean** | **IC 95%** | |
| --- | --- | --- | --- | --- | --- | --- | --- | --- |
|  |  |  |  | **P25** | **P75** |  | **Lower** | **Upper** |
| ATF4 | CQ | 11 | 0.92 | 0.64 | 1.03 | 1.10 | 0.57 | 1.63 |
|  | Not CQ | 25 | 0.81 | 0.47 | 1.06 | 0.78 | 0.63 | 0.92 |
| ATF6 | CQ | 16 | 0.35 | 0.16 | 0.87 | 0.64 | 0.23 | 1.04 |
|  | Not CQ | 28 | 0.19 | 0.11 | 0.27 | 0.27 | 0.15 | 0.38 |
| BIP | CQ | 9 | 1.50 | 1.08 | 1.89 | 1.80 | 0.82 | 2.78 |
|  | Not CQ | 22 | 1.59 | 0.94 | 2.21 | 1.66 | 1.32 | 2.00 |
| CALR | CQ | 11 | 0.53 | 0.25 | 0.96 | 0.62 | 0.33 | 0.92 |
|  | Not CQ | 27 | 0.56 | 0.29 | 0.98 | 0.63 | 0.47 | 0.78 |
| CANX | CQ | 12 | 0.75 | 0.23 | 0.97 | 0.87 | 0.37 | 1.38 |
|  | Not CQ | 26 | 0.88 | 0.19 | 1.36 | 0.87 | 0.61 | 1.13 |
| CHOP | CQ | 16 | 0.27 | 0.15 | 0.66 | 0.65 | 0.17 | 1.13 |
|  | Not CQ | 28 | 0.22 | 0.11 | 0.31 | 0.34 | 0.17 | 0.51 |
| PERK | CQ | 13 | 1.25 | 0.59 | 2.08 | 1.35 | 0.79 | 1.90 |
|  | Not CQ | 25 | 1.64 | 0.87 | 2.72 | 1.80 | 1.30 | 2.30 |
| XBP1 | CQ | 15 | 1.15 | 1.06 | 1.99 | 1.68 | 0.86 | 2.51 |
|  | Not CQ | 26 | 1.46 | 1.05 | 2.27 | 1.53 | 1.21 | 1.84 |

(*) Mann-Whitney test; IQR = interquartile range; IC 95% = confidence interval 95%
